# There's not always one enemy: Co‐infection of campylobacter jejuni and non‐typhoidal salmonella in a patient with systemic lupus erythematosus

**DOI:** 10.1002/ccr3.6515

**Published:** 2022-11-15

**Authors:** Daisuke Asatori, Kota Shimada

**Affiliations:** ^1^ Department of Rheumatic Diseases Tokyo Metropolitan Tama Medical Center Tokyo Japan

**Keywords:** campylobacter, co‐infection, immunocompromised patient, salmonella, systemic lupus erythematosus

## Abstract

A 22‐year‐old, female patient with systemic lupus erythematosus experienced bacterial enteritis. A stool Gram stain revealed Campylobacter. Non‐typhoidal Salmonella was detected in a stool culture, and Campylobacter jejuni was detected in a blood culture. Based on these findings, co‐infection of Campylobacter jejuni and non‐typhoidal Salmonella was diagnosed.

## Abstract 2

Because bacterial enteritis in immunocompromised patients can involve a co‐infection, thorough evaluation consisting of a stool Gram stain, stool culture, and blood culture is advisable.

## 1 INTRODUCTION

In community‐onset, infectious enteritis, the incubation period and exposure history may serve as clues to identifying the causative organism, but a stool Gram stain and stool culture are necessary for certain identification. Furthermore, judicious use of antimicrobial agents should be considered as immunocompromised individuals, elderly patients, and patients with an internal prosthetic device have an increased risk of serious illness and complications.^1^ When bacterial enteritis is suspected, involvement of Campylobacter or non‐typhoidal Salmonella is often suspected, and the initial empirical treatment usually targets one or the other. We herein reported a case of bacterial enteritis caused by a co‐infection of *Campylobacter jejuni* and non‐typhoidal Salmonella.

## 2 CASE PRESENTATION

A 22‐year‐old female patient with systemic lupus erythematosus (SLE) receiving prednisolone 4 mg, tacrolimus 2 mg, hydroxychloroquine 300 mg, and belimumab 200 mg weekly presented to the emergency department with headache and chills of 2 days' duration and vomiting, non‐bloody, watery stools, abdominal pain, and fever of 1 day's duration. History taking revealed that she had eaten grilled chicken 5 days earlier. The time of symptom onset and the history of food intake led to the suspicion of Campylobacter or non‐typhoidal Salmonella infection. A stool Gram stain and stool culture were performed; the former revealed Gram‐negative spiral rods, leading to the diagnosis of Campylobacter enteritis. As the patient was immunocompromised, azithromycin 500 mg was administered for 3 days. The abdominal pain and vomiting improved thereafter, and the fever gradually resolved. Later, a stool culture grew non‐typhoidal Salmonella. Because the patient was able to resume oral food intake and her general condition improved markedly, she was discharged on hospital Day 5. Four days later, *Campylobcter jejuni* was detected in a blood culture performed on the day of admission, and the patient was asked to return to the outpatient clinic. A second blood culture was performed, and an additional 14 days of azithromycin therapy was prescribed. The second blood culture remained negative after 7 days, and there was no symptom flare after the azithromycin therapy.

## 3 DISCUSSION

In the present case, both Campylobacter and non‐typhoidal Salmonella were considered the causative microorganisms on the basis of the incubation period and exposure history. The incubation periods of Campylobacter (2–5 days) and non‐typhoidal Salmonella (1–3 days) overlap. Chicken meat, which was eaten by the patient prior to symptom onset, also sometimes harbors both Campylobacter and non‐typhoidal Salmonella causing infectious enteritis.^1^

Bacterial enteritis caused by a co‐infection of Campylobacter and non‐typhoidal Salmonella was diagnosed in the present patient. Since the specificity of a stool Gram stain for Campylobacter is about 99%,^2^ the Gram‐negative spiral rods indicated a very high probability of Campylobacter enteritis. At the same time, non‐typhoidal Salmonella was detected in a stool culture, and the clinical symptoms were consistent with non‐typhoidal Salmonella enteritis. In the present case, Campylobacter was detected in the blood culture and stool Gram stain but not in the stool culture, in line with a finding that in rare cases, a Gram stain can be positive for Campylobacter even when a culture is negative,^2^ suggesting that a stool Gram stain may be useful for diagnosing Campylobacter enteritis microbiologically.

The present patient was susceptible to *Campylobacter jejuni* bacteremia because she had immunocompromised status, a known risk factor of *Campylobacter jejuni* bacteremia, and was receiving glucocorticoids and immunosuppressants.^3^ The number of immunocompromised patients has been on the rise in recent years for a variety of reasons, such as more intensive immunosuppressive therapy and an aging demographic. We may therefore expect the incidence of *Campylobacter jejuni* bacteremia to increase as well.

*Campylobacter jejuni* bacteremia should not be treated as transient in immunocompromised patients. In general, *Campylobacter jejuni* bacteremia is associated with a mortality rate of 0%–17%, and its prognosis is relatively good,^4^ with some patients improving spontaneously even without antimicrobial therapy.^5^ However, the mortality rate is higher in immunocompromised patients who do not receive antimicrobial therapy.^6^ Although some experts consider *Campylobacter jejuni* bacteremia to be a category of transient bacteremia that does not require treatment, the present patient was immunocompromised, justifying the use of antimicrobial therapy.

Choosing the optimal antimicrobial therapy for treating *Campylobacter jejuni* bacteremia can be difficult owing to the lack of high‐quality evidence based on studies comparing the efficacy of various treatments. A variety of antimicrobial treatments are used, including ampicillin, ceftriaxone, ciprofloxacin, levofloxacin, meropenem, erythromycin, azithromycin, and fosfomycin, and a standardized treatment period has not been established because some cases of transient bacteremia resolve without treatment..^4^, ^7^ Reports of past treatments have shown a considerable variation in the duration of treatment (4–19 days) and choice of antimicrobial agents. Many, previous patients were not treated with macrolides.^7^, ^8^ Among those that were, one received erythromycin for 12 days.^8^

Non‐typhoidal Salmonella is usually expected to resolve spontaneously without antimicrobial therapy.^9^ However, antimicrobial therapy against non‐typhoidal Salmonella is recommended for immunocompromised patients.^10^ On the contrary, antimicrobial therapy is also thought to increase the rate of bacterial carriage.^9^ Ceftriaxone and fluoroquinolones are recommended for the treatment of non‐typhoidal Salmonella,^10^ but our patient improved without either of these antimicrobials. The 14 additional days of azithromycin therapy in the present case led to a favorable clinical course.

## 4 CONCLUSION

The causative microorganisms in enteritis are clinically difficult to identify, and a co‐infection of *Campylobacter jejuni* and non‐typhoidal Salmonella may occur, as the present case has shown. Therefore, whenever bacterial enteritis is suspected in an immunocompromised patient, it is important to perform blood and stool cultures and, whenever possible, a stool Gram stain as well.

## AUTHOR CONTRIBUTIONS

Daisuke Asatori was involved in the literature search wrote the clinical report. Kota Shimada revised the manuscript.

## CONFLICT OF INTEREST

The authors have no conflicts of interest to declare.

## ETHICAL APPROVAL

None.

## CONSENT

Written informed consent was obtained from the patient to publish this report in accordance with the journal's patient consent policy.

## Data Availability

No Supplement.
